# Small bowel obstruction and strangulation secondary to an adhesive internal hernia post ESWL for right ureteral calculi: a case report and review of literature

**DOI:** 10.1186/s12876-021-01760-2

**Published:** 2021-04-17

**Authors:** Elaine N. Gitonga, Haitao Shen

**Affiliations:** Department of Emergency Medicine, Shengjing Hospital of China Medical University, Shenyang, 110004 People’s Republic of China

**Keywords:** ESWL, Adhesion, Internal hernia, Adhesive internal hernia, Small bowel obstruction

## Abstract

**Background:**

Extracorporeal shock wave lithotripsy (ESWL) is a relatively safe and convenient mode of treatment for ureteral and renal stones, despite its relative safety; ESWL is not without its complications. We present a case of a patient we managed for small bowel obstruction and strangulation due to an adhesive internal hernia after ESWL was done because of right ureteral calculi.

**Case presentation:**

We report a case of a 59-year-old patient who presented with severe abdominal pain a few hours after ESWL because of a right upper ureteric calculus. The abdominal pain increased in severity in time and became more generalized. The patient had one episode of gross hematochezia as she was being prepped for emergency laparotomy. Intra-op, she had a strangulated internal hernia because of an omental-mesenteric adhesion.

**Conclusion:**

This case report hopes to highlight the potential of complications like acquired IH due to adhesions in patients with a history of ureteral calculi, and also the complications that may come about post-ESWL. Patients who present with signs of persistent abdominal pain post-ESWL should be vigilantly observed. If symptoms persist, increase in intensity or there is a general deterioration of the patients’ hemodynamic status, even in light of negative MDCT findings, prompt surgical intervention is crucial for definitive diagnosis as well as management.

## Background

Extracorporeal shock wave lithotripsy has advanced from its invention in the 1980s to becoming a relatively safe and non-invasive method used in treating renal, ureteral, and biliary stones. ESWL applies focused shock waves to the stones; the force applied by the shock waves brings about disintegration and destruction of calculi within the urinary and biliary system [1, 25]. Despite its relative safety, ESWL is not without its complications [2]. ESWL-related complications can be described as renal and extra-renal. Renal complications include urosepsis, hematuria, and outflow obstruction by the stone fragments, reduced renal function, and systemic hypertension [3, 6]. The most common extra-renal complications of ESWL are to the gastrointestinal system, with fewer cases causing pulmonary complications [6–14]. These extra-renal complications come about from both the shear stress and the cavitation process created by the shock wave pulses essential in the pulverization of the stones. Multiple cases of gastrointestinal complications after ESWL have been documented, but we are still unaware of any documented case of small bowel strangulation because of an adhesive internal hernia post ESWL. We, therefore, present a case of a patient we managed for small bowel obstruction and strangulation due to an adhesive internal hernia after ESWL was done because of right ureteral calculi. Diagnosis of the IH was missed in the MDCT done. The final diagnosis of adhesive IH was found intra-op after the patient was taken in for emergency exploratory laparotomy.

## Case presentation

A 59-year-old female presented at our emergency department with complaints of severe abdominal pain several hours after undergoing ESWL in another hospital. She presented at the peripheral hospital with her chief complaint being an acute onset abdominal pain that started 4 h before arriving at the hospital. At the time the abdominal pain was intermittent, localized (mainly around the umbilical region), and radiated to the right flank. She also complained of nausea and had one episode of vomiting. After evaluation, an abdominal CT-Scan and abdominal ultrasound both revealed a right ureteral calculus and right renal hydronephrosis. They then scheduled her for ESWL. Unfortunately, in the patient's discharge summary, they did not disclose the specifics of the ESWL in terms of the shock waves delivered and the power range. The procedure was uneventful, and they discharged the patient.

After some hours, the patient started complaining of diffuse abdominal pain. She was then referred to our hospital for further management. At our facility, she presented with complaints of diffuse abdominal pain with associated nausea and vomiting. She did not report any dysuria, change in bowel movements, or hematochezia prior to the ESWL. Before this admission, her past medical history was uneventful; she had no history of any chronic illnesses, significant abdominal trauma, or abdominal surgery.

On physical examination, she was tachycardic but normotensive. Her abdomen was distended with generalized abdominal tenderness and mild rebound tenderness, but with no guarding present. Murphy's sign was negative. There was tenderness on percussion of the right kidney. Bowel sounds were increased at around 5/min and positive shifting dullness was present. Her other systems were normal.

Routine blood tests found that she had raised WBC at 26.9 ↑ 10^9/L^ and absolute neutrophilia of 76.5%, HB at 128 g/L, and hematocrit of 36.8%. CRP was elevated at 36.2 mg/L, revealing an inflammatory picture. LFT's showed a decrease in albumin at 26.8 g/L. Urea and creatinine were elevated at 9.2 mmol/L and 98.7 mmol/L respectively. Her coagulation profile was deranged, with prothrombin time and d-dimers at 12.6 s and 1302 μg/L, respectively. Urinalysis, urinary occult blood of 3 + , urinary protein 2 + , RBC 459.32/HP, WBC 45.83/HP.

After surgical consultation, we immediately scheduled her for an emergency exploratory laparotomy. As the patient was being prepared for surgery, she had one episode of hematochezia of approximately 300mls of fresh blood. Because of this, she received a transfusion of four units of packed red blood cells.

The laparotomy revealed that there was a large hematocele, with partial necrosis of the small intestine. The small intestine was entrapped by omental and mesenteric adhesions approximately 2 m from the ligament of Treitz. The bowel segments entrapped within the adhesions were released. Unfortunately, assessment of the bowel segment revealed that it was necrotic. The 2.6 m of the necrotic intestinal segment was resected and the continuity of the remaining 40 cm of the ileocecum and jejunum was maintained by end-to-end anastomosis.

## Outcome and follow up

The patient’s postoperative course was uneventful. She was discharged after four days and was to be followed up at the urology and surgical outpatient clinic.

## Discussion and conclusion

Adhesions are a form of vascularized scar tissue that brings about a connection or adherence between surfaces and organs within the peritoneal cavity. They result from a pathological healing process in response to a peritoneal insult or injury. The main causes of abdominal adhesions are inflammation, surgeries, intraperitoneal infections, radiation, and also significant abdominal trauma [15, 16]. Any peritoneal insult causing mesothelial injury causes an acute regional inflammatory which in turn increases capillary permeability and therefore leakage of blood from the surrounding capillaries. This encourages the accumulation of fibrinogen-rich exudate that is cleaved into a fibrinous matrix, that later disintegrates to promote healing through fibrinolysis. For adhesions, the fibrinous matrix persists and is stabilized through the organization processes (cellularization, vascularization, and innervation), creating a mature adhesion [29].

Adhesions have been linked to being a major cause of acquired internal hernias, and an increased risk of mechanical small bowel obstruction [17], 18. The severity of presenting symptoms in IH is related to the duration and reducibility of the hernia. The symptoms range from non-specific mild epigastric pain to an acute abdomen. After a bowel segment is entrapped within the IH, the normal luminal flow gets obstructed causing dilatation of the proximal segment and an increase in the intraluminal pressure of the distal segment. As the process continues the intestinal wall becomes edematous which leads to the transudative loss of fluid into the peritoneal cavity. As the intraluminal pressure reaches systolic blood pressure, the blood supply to the intestine becomes completely compromised leading to small bowel incarceration, strangulation, and finally necrosis [19].

Since the clinical diagnosis of internal hernias still proves to be difficult, the mainstay modality of choice for the diagnosis of IH to date is the multi-detector CT-scan (MDCT). The most specific MDCT findings or key features in relation to adhesive IH include; clustering or crowding of intestinal segments, crowding of mesenteric blood vessels, kinking or angulation of bowel loops, bowel wall thickening, presence of a hernia orifice, and the fat notch sign. Mesenteric infiltrates, localized mesenteric fluid and asymmetric bowel wall thickening are indicators of intestinal strangulation [20, 21]. Despite these key features, internal hernias are still misdiagnosed. Failed or erroneous diagnoses are usually because of two main factors: failure of the radiologist in recognizing the CT findings of internal hernias and also despite the high specificity and sensitivity of MDCT, up to 20% of cases of adhesive IH have negative CT findings [21, 22].

Since its inception of ESWL in the 1980s, it has developed into a relatively safe and non-invasive treatment for nephrolithiasis and has an overall stone-free rate of 75%. ESWL delivers focused high-energy pressure pulses consisting of a short duration initial phase of positive pressure pulse (P +) and a relatively prolonged negative pressure phase (P-) through the aid of a lithotripter. The difference in pressure gradient between P + and P- plays different roles in the fragmentation process of calculi through four fundamental mechanisms; shear force, cavitation, quasi-static squeezing, and dynamic fatigue [23–26, 28]. These important mechanisms are also responsible for tissue damage, especially if there is an underlying pathology that compromises tissue strength. ESWL induced tissue injury is primarily because of the effect of the shock wave pulses on the tissues themselves or from cavitation-induced tissue or vascular injury.

For our patient, in relation to the course of events and the appearance of abdominal symptoms, she probably had a self-reducible adhesive internal hernia that came about from a localized inflammatory process i.e. the ureteral calculi, since all the other causes of adhesive intestinal hernias were ruled out. The intestinal loop entrapped by the adhesion was along the pathway of the energy pulses, therefore causing an alteration in the acoustic impedance during the transmission of the shock wave. This change caused the dissipation of part of the high-energy pulses and initiation of the cavitation process on the surrounding tissue [27]. This expedited the small bowel tissue damage precipitating strangulation and necrosis of the small intestines.

**Fig. 1:**
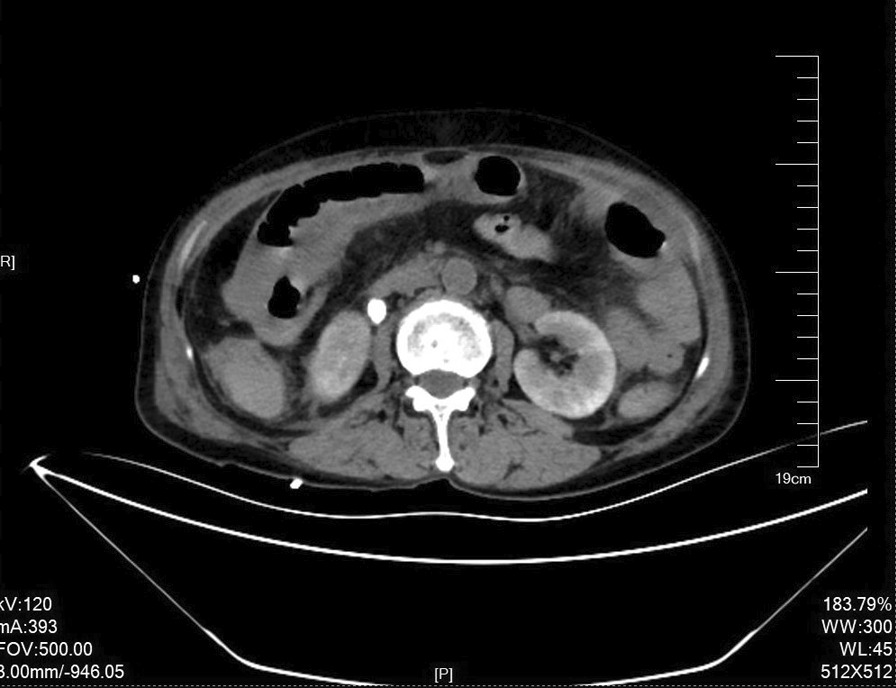
Abdominal CT-scan: intestinal dilatation, intestinal wall edema, and mesenteric exudation. Free fluid in the abdominal and pelvic cavity. Right lateral upper ureteric calculi measuring approximately 9 mm * 10 mm with right upper ureteral dilatation and hydronephrosis

In conclusion, despite ESWL being a relatively safe and effective method of treatment for nephrolithiasis, as clinicians, we should be aware of the possibility of acquired IH in patients with a history of ureteral calculi and the complications associated with it. Patients who present with persistent abdominal pain post-ESWL should be vigilantly observed. If symptoms persist, increase in intensity or there is a general deterioration of the patients’ hemodynamic status, even in light of negative or inconclusive MDCT findings, prompt surgical intervention is imperative for definitive diagnosis as well as management in order to avoid further morbidity and also reduce any chances of mortality (Fig. 1).

## Data Availability

The images and electronic medical records are available for review upon request, but the data will not be shared to protect the patient’s confidentiality.

## Abbreviations

ESWL: Extracorporeal shock wave lithotripsy
IH: Internal hernia
MDCT: Multidetector CT-scan

## References

[1] Taylor MC, Marshall JC, Fried LA, LeBrun GP, Norman RW (1988). Extracorporeal shock wave lithotripsy (ESWL) in the management of complex biliary tract stone disease. Ann Surg.

[2] D’Addessi A, Vittori M, Racioppi M, Pinto F, Sacco E, Bassi P (2012). Complications of extracorporeal shock wave lithotripsy for urinary stones: to know and to manage them-a review. ScientificWorldJournal.

[3] Nasu Y, Kurashige T, Kumon H, Akimoto M, Higashihara E, Kumon H, Masaki Z, Orikasa S (2001). Common and uncommon complications related to ESWL. Treatment of urolithiasis recent advances in endourology.

[4] McAteer JA, Evan AP (2008). The acute and long-term adverse effects of shock wave lithotripsy. Semin Nephrol.

[5] Silberstein J, Lakin CM, Kellogg PJ (2008). Shock wave lithotripsy and renal hemorrhage. Rev Urol.

[6] Kim TB, Park HK, Lee KY, Kim KH, Jung H, Yoon SJ (2010). Life-threatening complication after extracorporeal shock wave lithotripsy for a renal stone: a hepatic subcapsular hematoma. Korean J Urol.

[7] Klug R, Kurz F, Dunzinger M, Aufschnaiter M (2001). Small bowel perforation after extracorporeal shockwave lithotripsy of an ureter stone. Dig Surg.

[8] Castillon F, Gonzalez-Enguita, and Vela-Navarrete,  (1999). Colonic perforation after extracorporeal shock wave lithotripsy. BJU Int.

[9] Karakayali F, Sevmiş Ş, Ayvaz İ, Tekin İ, Boyvat F, Moray G (2006). Acute necrotizing pancreatitis as a rare complication of extracorporeal shock wave lithotripsy. Int J Urol.

[10] Willekens I, Brussaard C, Raeymaeckers S, De Concinck V, de Mey J (2015). Splenic rupture: a rare complication of extracorporeal shock wave lithotripsy. J Belgian Soc Radiol.

[11] Chhor V, Sinaceur M, Journois D (2009). Misleading abdominal pain following extracorporeal renal lithotripsy. Urol Int.

[12] Gupta S, Scambia J, Gandillon C, Aversano F, Batista R (2016). Abdominal compartment syndrome and necrotizing pancreatitis following extracorporeal shock wave lithotripsy. Urol Case Rep.

[13] Mokhtari M, Kumar PV, Ghayumi MA (2013). Eosinophilic pleural effusion: a rare complication of extracorporeal shock wave lithotripsy. Case Rep Med.

[14] Samkaoui MA, Ziadi A, Harifi G, Rhassan El Adib A, Younous S (2009). Contusion pulmonaire sévère secondaire à une lithotritie extracorporelle. Ann Fr Anesth Réanim.

[15] Tabibian N, Swehli E, Boyd A, Umbreen A, Tabibian JH (2017). Abdominal adhesions: a practical review of an often overlooked entity. Ann Med Surg (Lond).

[16] Liakakos T, Thomakos N, Fine PM, Dervenis C, Young RL (2001). Peritoneal adhesions: etiology, pathophysiology, and clinical significance. Dig Surg.

[17] Maciver AH, Michael McCall AM, Shapiro J (2011). Intra-abdominal adhesions: cellular mechanisms and strategies for prevention. Int J Surg.

[18] Akyildiz H, Artis T, Sozuer E, Akcan A, Kucuk C, Sensoy E, Karahan I (2009). Internal hernia: complex diagnostic and therapeutic problem. Int J Surg.

[19] Martin LC, Merkle EM, Thompson WM (2006). Review of internal hernias: radiographic and clinical findings. Am J Roentgenol.

[20] Yen CH, Chen JD, Tui CM, Chou YH, Lee CH, Chang CY, Yu C (2005). Internal hernia: computed tomography diagnosis and differentiation from adhesive small bowel obstruction. J Chin Med Assoc.

[21] Dou L, Yang H, Wang C (2019). Adhesive and non-adhesive internal hernia: clinical relevance and multi-detector CT images. Sci Rep.

[22] Lu C-Y, Xu M, Lin J, Chen Y, Gao Y, Wang Z-F, Zhao Z-W, Song J-J, Que H-F, Ji J-J (2018). Adhesive internal hernia: multidetector CT findings and clinical relevance. Clin Radiol.

[23] Junuzovic D, Prstojevic JK, Hasanbegovic M, Lepara Z (2014). Evaluation of extracorporeal shock wave lithotripsy (ESWL): efficacy in treatment of urinary system stones. Acta Inform Med.

[24] Coptcoat MJ, Webb DR, Kellett MJ (1987). The complications of extracorporeal shockwave lithotripsy: management and prevention. J Urol.

[25] Tailly GG (2013). Extracorporeal shock wave lithotripsy today. Indian J Urol.

[26] Eisenmenger W (2001). The mechanisms of stone fragmentation in ESWL. Ultrasound Med Biol.

[27] Zhong P, Zhou Y, Zhu S (2001). Dynamics of bubble oscillation in constrained media and mechanisms of vessel rupture in swl. Ultrasound Med Biol.

[28] Cleveland RO, Mcateer JA (2012). Physics of shock-wave lithotripsy. Smith's textbook of endourology.

[29] Cheong YC, Laird SM, Li TC, Shelton JB, Ledger WL, Cooke ID (2001). Peritoneal healing and adhesion formation/reformation. Hum Reprod Update.

